# Parliament and Publications

**Published:** 1911-04-08

**Authors:** 


### Parliament and Publications.

### RAG FLOCK.

Mr. John Burns has introduced in the House of Commons a Bill to prohibit the sale and use for the purpose of the manufacture of bedding, etc., of unclean flock manufactured from rags.

### PLAGUE IN INDIA.

In reply to Mr. Keir Hardy, Mr. Montagu stated that during the present cold weather plague in India had been more severe than in any of the last three years. Deaths last month numbered 88,498.

### SMALL-POX AND HOSPITAL ACCOMMODATION.

In reply to a question by Mr. Aster, Mr. John Burns stated that some 2,000 beds are available at short notice for small-pox cases in London, and this number could be largely increased if necessity arose.

### HEALTH VISITORS.

A Bill has been introduced in the House of Commons by the President of the Local Government Board to enable Local Authorities under the Notification of Births Act to appoint visitors for the purpose of giving to persons advice as to the proper nature, care, and management of children under five years of age.

### DENTISTS' MOTOR-CARS.

The relief allowed in respect of motor-car licence duty and motor-spirit duty to duly qualified medical practitioners is not applicable to dentists. With reference to this matter Mr. Hobhouse has pointed out that the saving of human life and suffering frequently depends upon a medical man possessing an expeditious means of locomotion, and in this respect the case of a dentist is not parallel.

### ABOLITION OF VIVISECTION BILL.

The Bill to provide for the abolition of vivisection was introduced by Mr. Lansbury. It proposes to make it unlawful to perform on any live animal, with or without the use of anæsthetics, any experiment, or demonstration, or inoculation of a nature to give pain or suffering, either directly or in its after-effects, for any pathological, surgical, physiological, or other scientific purpose, or for the acquirement of manipulative skill.

### DISEASES OF ALIENS.

Leave to land under the Aliens Act was refused to 623 immigrants during the years 1909 and 1910, the particular grounds of rejection being as follows: Trachoma, 299; other eye diseases, 25; venereal diseases, 90; skin and scalp diseases, 25; lunacy or idiocy, 5; tubercular diseases, 8; miscellaneous diseases, 10; infirmities likely to lead to chargeability (including cardiac disease, deformity, senile decay, paralysis, etc.), 161.

### NOTIFICATION OF TUBERCULOSIS.

The Local Government Board have issued an Order embodying further regulations to provide for the notification of cases of pulmonary tuberculosis occurring amongst the in-patients or out-patients at hospitals, or other similar institutions for the treatment of the sick, which are supported wholly or partly otherwise than by the contributions of the patients (or of their relatives or guardians) and otherwise than from rates and taxes. By this Order the medical officer who is in medical attendance upon the patient at the hospital is required to notify the Medical Officer of Health for the area in which the hospital is situated; the notification must be sent within forty-eight hours after the first recognition of the disease. The remuneration of medical officers who are required to make notifications under the order is fixed at one shilling. The Board are aware that a correct dignosis of pulmonary tuberculosis involves the occasional need for bacteriological examination of the sputum. It may be pointed out, however, that no new duty is imposed by the Order upon the officers of hospitals beyond that of transmitting in a prescribed form information which is acquired in the ordinary course of professional attendance. The Order will come into operation on May 1.

